# Clinical practice for outpatients that are chronically red cell dependent: A survey in the Netherlands

**DOI:** 10.1111/vox.13220

**Published:** 2021-12-12

**Authors:** Rik P. B. Tonino, Martin R. Schipperus, Jaap Jan Zwaginga

**Affiliations:** ^1^ Haematology LUMC Leiden The Netherlands; ^2^ Haematology Haga Teaching Hospital The Hague The Netherlands; ^3^ Research TRIP Haemovigilance and Biovigilance Office The Hague The Netherlands; ^4^ CTCR Sanquin Blood Supply Leiden The Netherlands

**Keywords:** chronic anaemia, patient blood management, red blood cell transfusion, survey, transfusion dependency

## Abstract

**Background and Objectives:**

Limited data are available to guide physicians on how to determine the red blood cell (RBC) transfusion regimen in chronically transfusion‐dependent patients. The lack of clarity on thresholds and targets to be used for transfusion could easily result in either under or over transfusion in these patients. The aim of our survey is to investigate (1) transfusion thresholds; (2) number of RBC units given per transfusion episode; (3) interval between transfusions and (4) patient factors, like decreased cardiac function modulating the former.

**Materials and Methods:**

We sent a web‐based 44‐question survey to members of the Dutch Haematology Association.

**Results:**

Fifty physicians responded between June and October 2020 (response rate 30%), well‐distributed between community and academic hospitals. A wide variation in transfusion strategies was reported: Most patients have transfused 1–2 RBC units (range: 0–3 units) every 2–4 weeks (range: 1–12 weeks) with a median threshold of 8.0 g/dl ranging from 6.4 to 9.6 g/dl. Patient‐specific clinical factors that are most frequently reported to influence the transfusion strategy are angina pectoris, cardiac failure and dyspnoea, softer parameters that are of influence are the quality of life and self‐sustainability.

**Conclusion:**

The results of this survey indicate a broad variation in RBC transfusion strategies in Dutch patients with chronic transfusion dependency. While the current variation in transfusion strategies may be unavoidable in an individualized approach, randomized trials and better defined usable parameters to evaluate the effect of transfusion strategies are required to reach a consensus on how to determine the transfusion strategy.

**Highlights**

- Great variability in transfusion strategies for chronic anaemic patients was found.
- Parameters associated with changes in transfusion strategies, like angina pectoris and self‐sustainability, are identified.
- The outcomes allows professionals to benchmark their transfusion strategies and practices and reflect on them, and can be used in training.

## INTRODUCTION

Patients who receive red blood cell (RBC) transfusions on a regular basis typically suffer from diseases like myelodysplastic syndrome (MDS), myeloproliferative diseases (MPN), thalassaemia, sickle cell disease or aplastic anaemia. Although these patients receive 20%–30% of all RBC transfusions in Europe [1, 2], limited data are available to guide physicians on how to determine a chronic RBC regimen [3, 4, 5, 6, 7]. As studies on restrictive RBC transfusion strategies show no disadvantages in other patient categories [8, 9, 10, 11, 12], physicians tend to use a restrictive transfusion policy based on the perceived Quality of Life (QoL) of the individual patient [13, 14, 15]. The effect of this individualized transfusion policy is hard to quantify and depends on the haemoglobin (Hb) threshold, the timing of transfusion and the number of RBC units administrated [6, 7, 16]. Factors like anaemia related symptoms, the (estimated) cardiopulmonary compensation capacity and the presence of iron overload might further influence the transfusion policy [17, 18]. The lack of clarity on thresholds and targets to be used for transfusion, will easily result in either under or over transfusion in these patients. Both moreover, will have negative effects, with on one hand anaemia‐related QoL [19, 20, 21, 22] and on the other iron‐overload and other transfusion‐specific side effects [23, 24, 25].

We performed a survey amongst haematologists in the Netherlands to investigate their current standard of care for patients dependent on chronic transfusions, regarding the applied (1) transfusion thresholds; (2) number of RBC units given per transfusion episode; (3) interval between transfusions and (4) patient factors, like decreased cardiac function modulating the former.

## MATERIALS AND METHODS

### Study design

In this cross‐sectional study, a structured, 44‐question online survey was conducted amongst members of the Dutch Society of Haematology (NVvH) that treat patients with chronic transfusion dependency due to a haematological disorder. The questionnaire was sent to 169 haematologists in the Netherlands (Appendix S1). The questions were related to both clinical and soft outcomes that might influence the transfusion policy, that is, the Hb threshold, amount of transfusions given and the interval between transfusions. In addition, respondents were asked to fill out a set of questions about one or more of their own transfusion‐dependent patients to test for consistency of actual practice with responses to similar general questions. To allow for quantitative answers, Likert scales were used [26]. Incomplete surveys were included in the analysis. Free‐text responses were interpreted and summarized by the authors. The study was conducted using a Castor Electronic Data Capture (Castor, Amsterdam, the Netherlands) based survey (Appendix S1).

### Statistical analysis

Categorical variables are summarized as frequencies and percentages or median with range and statistically evaluated with the chi‐square test, continuous variables are reported as means and standard deviations and were statistically evaluated with unpaired *t*‐test, Welch's *t‐*test and Pearson's correlation coefficient. Statistical methods were conducted using SPSS (version 25.0, SPSS Inc., Chicago, IL), *p*‐values <0.05 were considered significant.

## RESULTS

### Study cohort

We sent out a survey to 169 members of the NVvH in the Netherlands of which 50 physicians responded between June and October 2020 (response rate: 30%). A total of 56% of the responding physicians work in an academic medical centre, 44% in a community hospital. Seven out of eight academic centres in the Netherlands were represented in the responses. The physicians completed questions on a cumulative total of 58 transfusion‐dependent patients. Out of the respondents, 42/50 were haematologists, 6 were haematologists in training and 2 were specialists in internal medicine. Including the years in training, 12 respondents had <5 years of experience with transfusion‐dependent patients; 19 respondents between 5 and 10 years; 9 between 10 and 19 years and 10 respondents had >20 years of experience.

### General response

**FIGURE 1:**
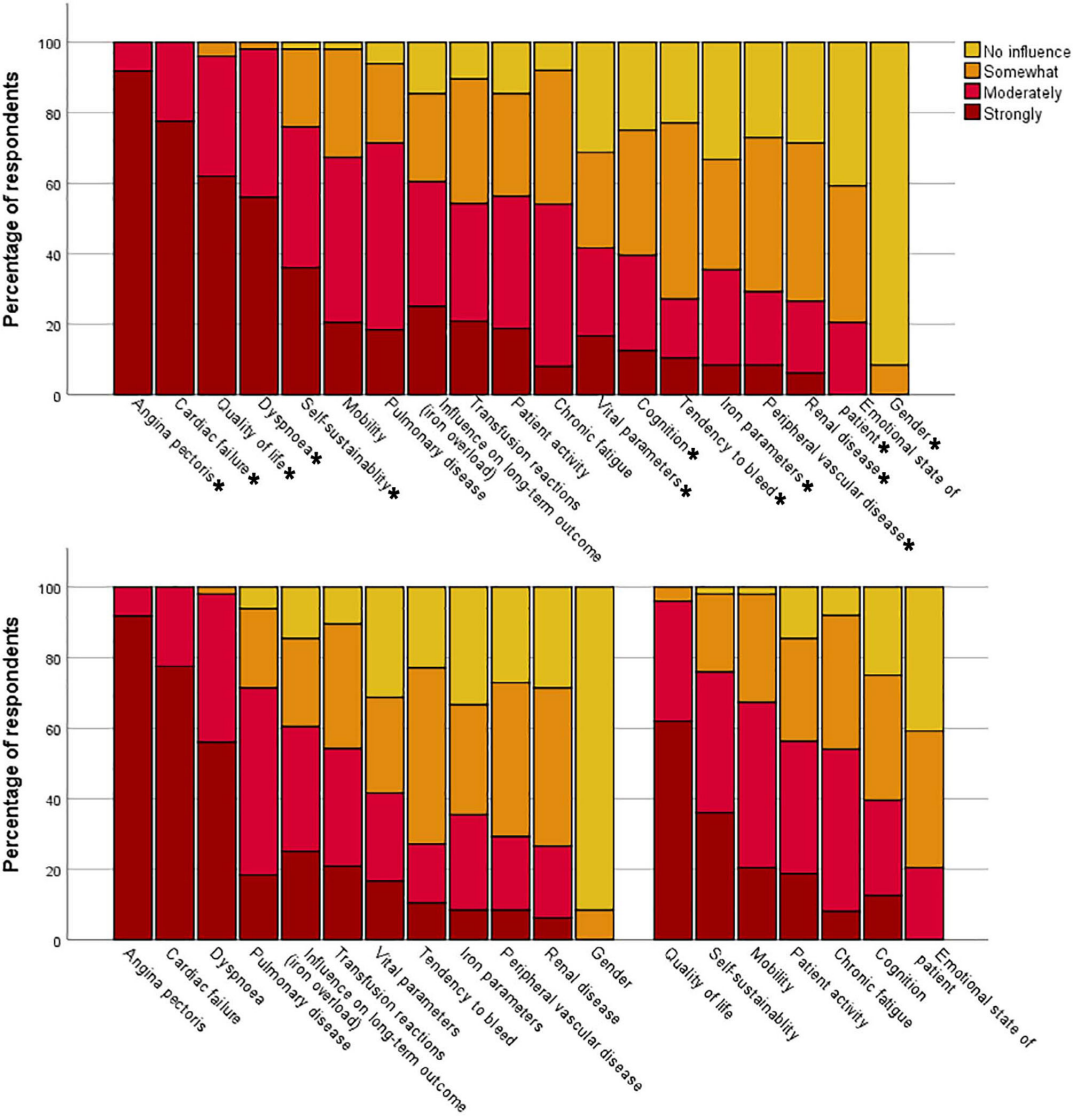
Responses to how much the indicated factors affect the choice for a red blood cell transfusion threshold, arranged from strongly to least influential (top); and subdivided in clinical and soft parameters (bottom). *p < 0.05 compared to the responses of all other parameters

In the first part of the survey, we asked physicians to indicate, which of a set of 19 given factors affects their transfusion strategy in general. In Figure 1, these factors are depicted. Clinical factors that are reported to strongly influence the transfusion threshold are angina pectoris, cardiac failure and dyspnoea. Soft outcomes that are reported to influence the threshold mostly are a decreased quality of life and self‐sustainability. Factors that are reported to be of no, or little, influence are gender, emotional state (fear and depression), renal disease, peripheral vascular disease, iron parameters and a bleeding tendency.

We stratified for years of experience of the haematologist and academic versus community hospital to evaluate for effect‐modification by these factors. While responses from academic‐ and community hospitals yielded comparable outcomes, the amount of experience of physicians did have influence on the weight they attributed to certain factors: patient activity, mobility and self‐sustainability were scored as ‘moderate‐strong influence on the Hb‐threshold’ in 8/9 (89%, *p* = 0.01), 10/10 (100%, *p <* 0.01) and 9/10 (90%, *p* = 0.17) by physicians with >20 years of experience, while physicians with <20 years of experience scored 17/39 (44%), 23/39 (59%) and 29/40 (74%), respectively.

Long‐term outcomes were taken into consideration more often by physicians with <5 years of experience: they scored a ‘moderate‐strong influence’ in 9/11 (82%) cases versus 20/37 (54%) for >5 years of experience (*p* = 0.07). Furthermore, 8/48 (17%) of the respondents (all of which have <10 years of experience) reported to measure post‐transfusion Hb‐levels.

### Patient‐specific response

**FIGURE 2:**
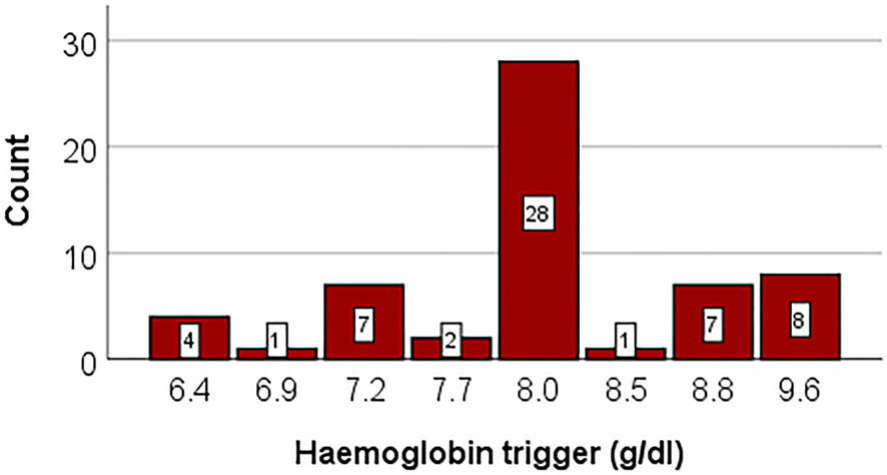
Haemoglobin thresholds used in daily practice (*n* = 58)

The reported Hb thresholds for RBC transfusion‐dependent patients are depicted in Figure 2. The most‐reported threshold (28/58; 48%) was 8.0 g/dl (range: 6.4–9.6 g/dl). Four patients received transfusions only when their Hb dropped below 6.4 g/dl, seven patients had a threshold of 7.2 g/dl. Sixteen patients had a threshold higher than the median, of these; eight were transfused when their Hb‐level dropped below 9.6 g/dl.

Regarding the transfusion interval: 7/56 patients (13%) received transfusions on a weekly basis, 11/56 patients (20%) every 2 weeks, 13/56 (23%) patients every 3 weeks, 13/56 (23%) patients every 4 weeks and only 12/56 (21%) patients have an interval of at least five or more weeks (Table 1).

**TABLE 1:** Overview of various individual transfusion regimes

	Number of RBC transfused		
	1 RBC, g/dl ± SD (*n*)	2+ RBCs, g/dl ± SD (*n*)	Total, g/dl ± SD (*n*)
Interval (weeks)			
1	8.3 ± 1.0 (4)	8.6 ± 0.9 (3)	8.4 ± 0.9 (7)
2–4	8.0 ± 0.7 (15)	8.3 ± 0.7 (22)	8.2 ± 0.7 (37)
5–12	8.9 ± 1.1 (2)	7.9 ± 1.2 (10)	8.1 ± 1.2 (12)
Total	8.1 ± 0.8 (21)	8.2 ± 0.9 (35)	8.2 ± 0.8 (56)

*Note*: A mean threshold is given per transfusion regime.

Abbreviation: RBC, red blood cell.

The number of transfused units was strongly correlated with the pre‐transfusion Hb‐level (*p* < 0.001). Table 2 shows the amount of RBCs transfused per episode, depending on how much the Hb‐level dropped below the patient's threshold. When the pre‐transfusion Hb is <0.8 g/dl below the patient's personal threshold, a mean of 1.6 RBC units was transfused. When the observed Hb‐level was between 0.8 and 1.6 g/dl below the threshold, a mean of 2.0 RBC units were transfused. When the Hb‐level was >1.6 g/dl below the threshold, the mean number of transfused units was 2.6.

**TABLE 2:** Amount of red blood cells (RBCs) transfused when the haemoglobin level drops <0.8, 0.8–1.6 or >1.6 g/dl below the patient's personal threshold

	Number of RBCs transfused per transfusion episode				
	0 RBCs, *n* (%)	1 RBC, *n* (%)	2 RBCs, *n* (%)	3 RBCs, *n* (%)	Cumulative count of transfused RBCs (*mean per patient*)
Hb‐level below the patients personal threshold					
<0.8 g/dl	1 (2%)	21 (36%)	34 (59%)	2 (3%)	95 (*1.6 pp*)
0.8–1.6 g/dl	0	8 (14%)	41 (71%)	9 (16%)	117 (*2.0 pp*)
>1.6 g/dl	0	0	26 (45%)	32 (55%)	148 (*2.6 pp*)

*Note*: A significant increase in transfused RBCs is seen with lower Hb‐levels (*p* < 0.001, for both <0.8 compared to 0.8–1.6 and >1.6 g/dl, and 0.8–1.6 compared to >1.6 g/dl).

Abbreviation: RBC, red blood cell.

**FIGURE 3:**
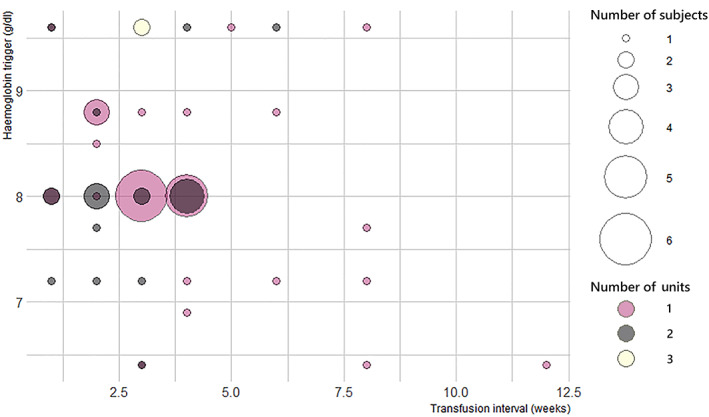
Bubble plot of the reported transfusion strategies. Most responses were focussed on a threshold of 8 g/dl every 2–4 weeks. Patients with thalassaemia and Sickle Cell Disease were reported to receive transfusions with a larger interval with also a lower mean threshold, which explains some of the outliers

The threshold, interval and number of units transfused together to form a transfusion strategy. The various transfusion strategies are cross‐tabulated in Table 1 and depicted in Figure 3. The mean threshold/(interval × transfusion amount) does not show a particular trend, suggesting that the variation in thresholds is independent of these two factors and more dependent on patient‐specific parameters. Although no single strategy prevails most, a common frame can be identified: 63% of the reported patients are transfused 1–2 units every 2–4 weeks.

### Parameters associated with changes in transfusion strategies

We asked the respondents to complete a set of questions on a patient of theirs to test for consistency with the general responses on the abovementioned factors. For patients without angina pectoris, the mean threshold was 8.0 g/dl whereas patients with moderate angina had a threshold of 8.8 g/dl (*p* = 0.047). This suggests that physicians indeed increase the transfusion threshold when a patient suffers from angina. Whether or not patients had any form of cardiac failure only did not impact the threshold in a significant manner (*p* = 0.28): a mean threshold of 8.1 g/dl for no cardiac failure versus 8.4 g/dl for patients with cardiac failure. Pulmonary disease had an, if any, inverse effect: 8.2 g/dl was the mean threshold for patients without the pulmonary disease, 8.0 g/dl for patients with pulmonary disease. The patient group without pulmonary disease did, however, have a higher prevalence of cardiac failure and angina pectoris.

The underlying disease, the cause for the patient's chronic anaemia, strongly impacts the transfusion strategy. While the overall median transfusion interval is 3 weeks, for 5/8 thalassaemia patients, the transfusion interval is 5 weeks or more (median = 6 weeks, range: 2–12, *p =* 0.038). Thalassaemia and sickle cell patients (*n* = 8 and 3, resp.) also have a lower transfusion threshold (7.2 ± 0.7; *p =* 0.001, and 7.5 ± 1.2 g/dl; *p* = 0.19, respectively). Other disease groups (MDS *n* = 22, MPN *n* = 13, AML *n* = 2, CMML *n* = 2, and other *n* = 7) all had a mean threshold between 8.2 and 8.6 g/dl with a median interval of 3 weeks (range: 1–8 weeks), except for one aplastic anaemia patient: threshold 9.7 g/dl, interval 1 week. Not all patients have a structured transfusion strategy: 2/3 sickle cell patients receive transfusions on demand.

Whether a patient received active treatment other than RBC transfusions for their underlying disease appears to affect the threshold too. The mean threshold for patients that received a form of chemotherapy was 7.6 ± 0.6 g/dl (*n* = 11, *p* = 0.02 compared to no treatment), while receiving immunomodulatory drugs leads to a threshold of 8.5 ± 0.6 g/dl (*n* = 8) and no treatment to 8.3 ± 0.9 g/dl (*n* = 38).

**FIGURE 4:**
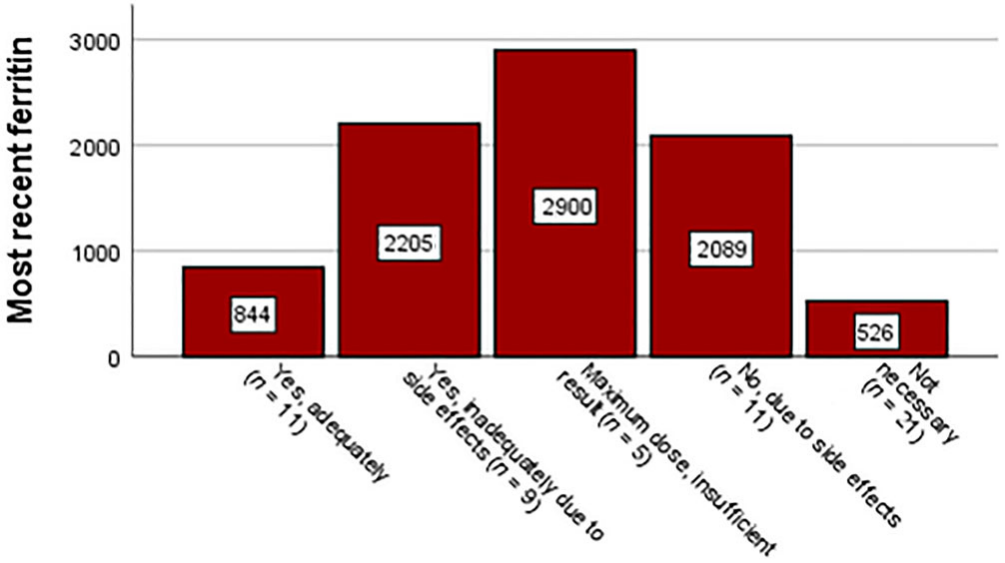
Mean ferritin per response to the question of whether the patient receives chelation therapy. The mean transfusion burden is given per response group. Patients that do not require chelation therapy have the highest mean transfusion burden (*p* = 0.042 compared to those who do receive chelation therapy)

Although most physicians report that they try to adjust transfusion strategies to avoid long‐term negative outcomes due to iron overload, they also report not necessarily taking iron parameters into the equation (Figure 2). Nonetheless, our data do show a mild effect of ferritin level on the threshold. When patients had a ferritin <1000 μg/L, the mean threshold was 8.3 ± 0.6 g/dl, while ferritin levels >1000 μg/L lead to a mean threshold of 8.0 ± 0.4 g/dl (*p =* 0.24). In addition, we evaluated the ferritin levels per response to the question ‘how much RBC units the physician would transfuse to their patients if the Hb‐level would drop more than 1.6 g/dl below their personal threshold’: the 32 patients that would receive three RBCs had a mean ferritin level of 1140 ± 944 μg/L while the 25 patients that would receive 2 RBCs had a mean ferritin level of 1647 ± 1948 μg/L (*p* = 0.20). Stratification for the transfusion burden yielded the same results. While the main cause for elevated ferritin levels is the transfusion burden, it is also dependent on how effective and/or tolerated chelation therapy is (Figure 4). Interestingly, the patients that were deemed not to need chelation therapy because of the still low mean ferritin levels did have the highest transfusion burden (2.9 units/4 weeks; *p* = 0.046 compared to patients that do receive chelation therapy). These three findings suggest that patients with higher ferritin levels are in fact transfused more restrictively.

Most parameters individually did not render an association with the transfusion threshold, interval or number of units transfused. However, trying to pinpoint what type of patients are most likely to deviate from the mean, we noticed that patients with a threshold <7.2 g/dl did not suffer from pulmonary or cardiac problems, and had few risk factors overall. Amongst patients with thresholds up to 8.0 g/dl, 3/14 (21%) suffered from cardiac and/or pulmonary problems, compared to 26/44 (59%) patients with thresholds above or equal to 8.0 g/dl (*p* = 0.014). This indicates that an important prerequisite for maintaining a maximally restrictive transfusion policy is sufficient (estimated) in cardiopulmonary compensation capacity. Conversely, as stated before, the presence of angina pectoris and cardiac failure may lead to a higher threshold. Other than that, we could not identify consistent motives for increasing the threshold above 8.0 g/dl.

Regarding physician factors, academic hospitals and community hospitals reported similar strategies. There are, however, some nuanced, non‐significant, differences: academic hospitals transfuse every 3.6 weeks (mean), while community hospitals transfuse every 4.4 weeks without a change in the mean transfusion threshold (both 8.2 g/dl). Physicians with <5 years of experience in the field appear to transfuse more units (1.8 units every 3.9 weeks) but at a more restrictive = lower threshold (8.0 g/dl), compared to colleagues with more experience, especially compared to those with >20 years of experience (1.6 units every 3.9 weeks, threshold 8.2 g/dl). We found no correlation between outliers in thresholds and the experience level of the physicians.

Because we included incomplete surveys, we performed a non‐response error analysis to evaluate whether bias might have been introduced by non‐responders. A relatively high amount of respondents failed to report the patient's most recent ferritin (5/58; 9%). We did not find a difference between those who did not report the ferritin level and those who did. Other questions were not left unanswered more frequently than others.

## DISCUSSION

This survey amongst Dutch haematologists shows an average transfusion strategy of 1–2 RBC units (range: 0–3 units) every 2–4 weeks (range: 1–12 weeks) with a median threshold of 8.0 g/dl but a large reported variation (range: 6.4–9.6 g/dl). Patient‐specific clinical factors that affected the found variability in the transfusion strategies are angina pectoris, cardiac failure and dyspnoea, but also ferritin levels, underlying disease and the concurrent treatment influenced transfusion regimen. Softer parameters of influence are quality of life and self‐sustainability. More experienced physicians take patient activity, mobility and self‐sustainability into account more often than less experienced colleagues.

Regarding the underlying disease, patients suffering from sickle cell disease and thalassaemia had a lower mean threshold (7.2 and 7.5 g/dl, resp.) and higher interval (median 6 weeks) compared to the other included patients (Hb‐threshold >8.2 g/dl, interval = 3 weeks). This may be explained by a lifelong need of these patients to cope with lower Hb‐levels but also by the known higher alloimmunization levels of these patients and thus more focus on restriction. In addition, transfusion goals differ between different indications: whereas an MDS patient receives transfusions to improve the oxygen‐transportation capacity, the aim in patients with sickle cell disease is also to decrease the proportion of sickle Hb relative to Hb A to prevent or reverse vaso‐occlusive disease.

Patients receiving chemotherapy are transfused at a lower mean threshold (7.6 g/dl) as compared to those with immunomodulatory drugs or no active therapy (8.5 and 8.3 g/dl, resp.). The suppression of haematopoiesis by chemotherapy, although more severe, is also more likely to be reversible. Physicians may thus accept a lower Hb level as a rise is to be expected when the chemotherapy's side effects wear off.

**FIGURE 5:**
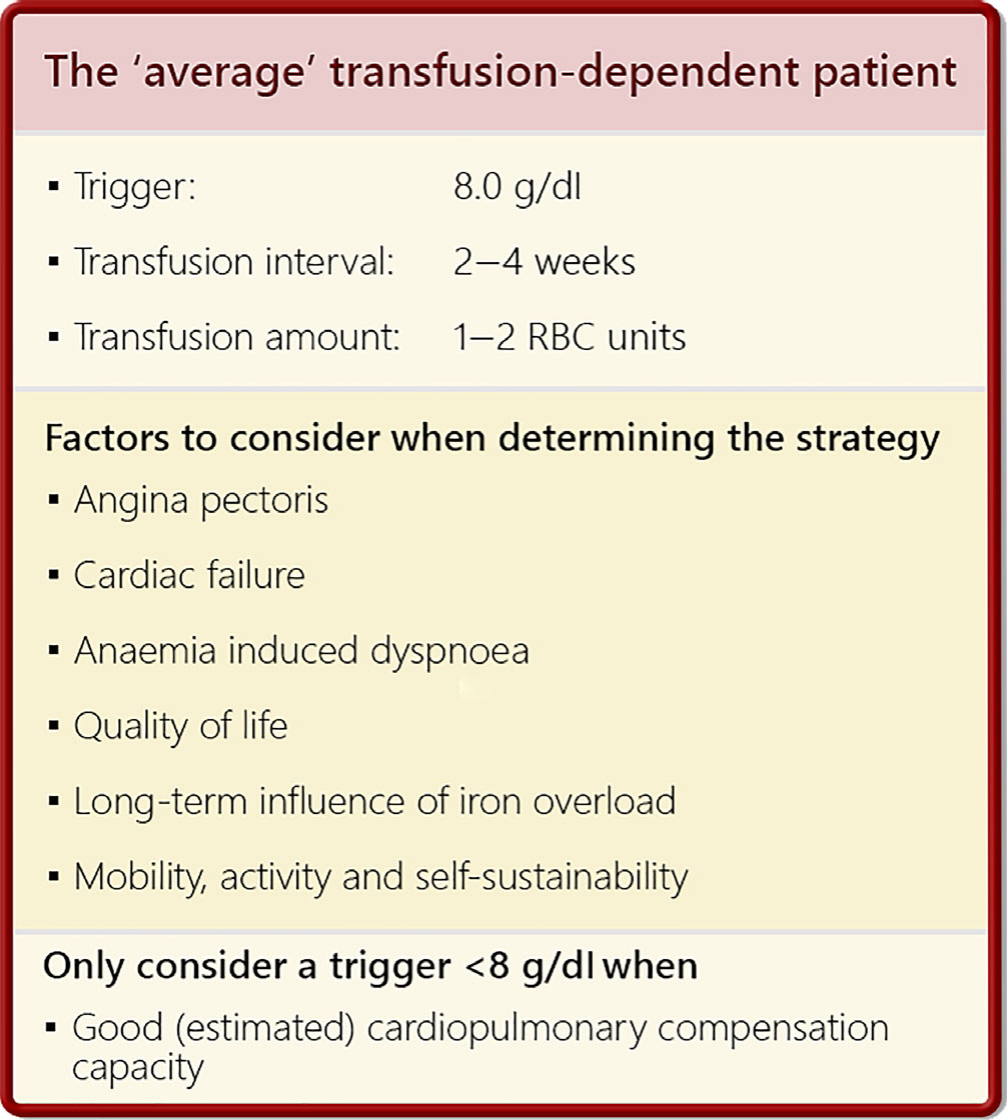
The average transfusion‐dependent patient according to the present survey. RBC, red blood cell

There may be various arguments to transfuse at a higher more liberal threshold: a physician might aim to increase the patient's QoL; or to counter a patient's poor tolerance of low Hb‐levels. Vice versa, restrictiveness with thresholds below 8 g/dL seems only enforced if there are no clear risk factors such as a reduced estimated cardiopulmonary compensation capacity (Figure 5).

Clearly, the exact effect of a transfusion on the patient is hard to quantify. As few physicians report to measure post‐transfusion Hb‐levels, this also appears to be a parameter of little value. Therefore, there is a dire need for clinically relevant outcome measures to evaluate transfusions and transfusion strategies.

Interestingly, an increased ferritin level did somewhat influence the threshold and transfusion burden, in spite of the fact that physicians score ferritin as one of the least influential factors. A possible relation indeed is not clear‐cut. Indeed, high ferritin levels could cause a more restrained transfusion strategy to avoid iron overload and consequent long‐term organ damage, while for patients with lower ferritin levels, a more liberal transfusion strategy could be allowed. On the other hand, the need for chronic transfusions is bound to lead to higher ferritin and iron overload, which could already urge the physicians to transfuse more restrictively as a precaution. Therefore, the observed but only moderated impact of elevated ferritin levels on the transfusion strategy might be an underestimation of iron overload awareness. In addition, whether chelation therapy is used, effective and/or tolerated further complicates this question, as it greatly affects ferritin levels. Assuming transfusion burden should be guided by the present or expected iron overload a new question could be raised: could patients in which chelation therapy is effective to be transfused at a higher threshold? The theoretical benefits of a higher Hb level, which have yet to be established, may indeed outweigh the side effects if they can be limited. In addition, an established gain in quality‐adjusted life years's might balance the extra labour and costs of transfusions. Clearly, not all variations of transfusion strategies can be accounted for by the investigated factors. Beside patient factors, the current variation in transfusion strategies is likely also due to lack of evidence‐based guidelines for this group of patients. Some physicians may base their choice to transfuse more restrictively on multiple studies conducted in other patient groups where restrictive RBC transfusion strategies show no disadvantages on the accumulated parameters like morbidity and mortality [8, 9, 10, 11, 12]. Others may choose a more liberal strategy because a higher Hb‐level is believed to be beneficial for the transfusion‐dependent patient [27]. While variability in transfusion strategies between patients may not be a problem at all, the lack of guidelines should not lead to large interphysician differences, as this will cause both under and over transfusion. Nevertheless, the observed range of thresholds does not find a base in Dutch or international guidelines [13, 28]. Currently, the Dutch guideline advises to use a threshold between 7.0 and 8.0 g/dl for reversible anaemia of haematological patients other than the chronically dependent group; importantly, its only advice for chronic anaemia is to apply an individual strategy based on the perceived quality of life.

This is the first survey amongst haematologists in the Netherlands in which important questions regarding RBC transfusion support in chronic transfusion‐dependent patients are addressed. Though some presented results may have been expected, it is important that they are documented in search of proper guidelines.

Regarding this paper's limitations, first, as is the case in most surveys, this study is limited by the moderate response rate. Aiming for respondents who take an interest in transfusion policies, and are, therefore, likely to treat a relatively high number of the low‐prevalent transfusion‐dependent patients, we believe, we have included a representative portion of the target population. Second, our data may be subject to response bias as physicians who do not maintain standard transfusion strategies, or who do not use transfusion thresholds may not have responded. Third, as only the responses of the physicians were collected, the actual clinical practice could differ. We should, for example, be cautious with the interpretation of ferritin levels, since we have no means of verifying these numbers. Fourth, because we included incomplete surveys, bias could have been introduced by non‐response errors. However, our non‐response error analysis suggests that this is not the case.

A survey looking into the transfusion thresholds of patients with haematological malignancies in general in the Netherlands yielded similar results [29]. However, the median threshold for outpatient care was lower—7.2 g/dl. Mo et al. performed a survey investigating thresholds for specifically MDS patients in Australia. In agreement with our results, they concluded a typical transfusion threshold of 8.0 g/dl, with higher thresholds typically used for patients with cardiovascular disease or anaemia symptoms [30]. In both the abovementioned surveys, the investigation of parameters associated with changes in transfusion strategies, however, was much more limited than the present study. Both these previous surveys reported on existing institutional transfusion guidelines and/or used case scenarios to elicit transfusion thresholds. The present study, in contrast, asked for actual patient examples, which might be a better reflection of the true spectrum of currently used transfusion strategies for chronic transfusion‐dependent patients. These new, original data could allow professionals to benchmark their transfusion practices and reflect on them, and maybe helpful in organizing haematologists' training.

The observed variation in less restrictive transfusion strategies ultimately stems from the variable use of non‐evidence‐based parameters to counter the cons of transfusions. While it is clear that fewer transfusions prevent transfusion reactions and iron overload, this results in a lower Hb‐level, which negatively impacts patients' well‐being [10, 19, 20, 21, 22]. Softer outcomes describing well‐being like health‐related quality of life, though reportedly important as shown by Figure 2, however, are harder to quantify.

Our findings hence address the need for prospective randomized trials to develop evidence‐based guidelines for RBC transfusions in patients with chronic transfusion dependency with the goal of improving their QoL while limiting unnecessary transfusions without compromising the outcome. To date, there have only been two small randomised controlled trials (13 and 38 patients) comparing restrictive versus liberal thresholds in chronic transfusion‐dependent patients. Although not adequately powered to detect clinically relevant differences, results are suggestive of an improved QoL with higher Hb‐levels [31, 32]. Currently, several studies with chronic transfusion‐dependent patients are conducted (REDDS‐2, ACTRN12619001053112; REMOTE 2, NL9289; EnhanceRBC, NCT02099669; SMD‐Transfu, NCT03643042; PTQA, NCT03660228). Results of these studies will yield better insight into the effects of transfusion strategies on the well‐being of this chronically transfused group of patients. We encourage international collaborations for trials investigating patients with chronic transfusion requirements. Such collaboration is likely required to achieve a large enough sample size to detect meaningful, clinically relevant differences in transfusion strategies.

In conclusion, the results of this survey indicate a broad variation in RBC transfusion strategies in Dutch patients with chronic transfusion dependency. While the current variation in transfusion strategies may be unavoidable in an individualized approach, randomized trials and better defined usable parameters, amongst, which a measure for QoL, to evaluate the effect of transfusion strategies are required to reach a consensus on how to determine the transfusion strategy.

## CONFLICT OF INTEREST

All authors attest that they have no conflict of interest to declare.

## Supporting information


**Data S1.** Supporting information.
