# Supplementary material for: Clinical practice for outpatients that are chronically red cell dependent: A survey in the Netherlands
Source: Vox Sang. 2021 Dec 12;117(4):526–34. doi: 10.1111/vox.13220 (PMC9299939; doi:10.1111/vox.13220)
Supplement: Supplementary file 1 — Data S1. Supporting information. [file VOX-117-526-s001.docx]

**Supplementary data**

**Survey**

Dear Colleague,

With no clear guidelines on how a chronic transfusion dependent patient should be transfused, we are curious about how the Dutch haematologist transfuses these patients.

Through this survey we wish to investigate the considerations of the haematologist, and create an overview of what currently is the standard care. We will be focusing on the haemoglobin trigger, number of units transfused, timing of transfusion, and patient factors affecting these parameters.

We will ask you in what centre you are employed in order to compare centres, the data will be anonymized. The survey will take 10-15 minutes to complete.

Thank you in advance for your cooperation

The original survey is in Dutch. This is a translated version.

- 1. In what center are you currently employed? ___________

1.2 City of center? ___________

1.3 What is your current function/specialty? ___________
□ Haematologist
□ Internal Medicine
□ In training
□ Other

1.4 What is your experience with Haematology
□ <5 years
□ 5-10 years
□ 11-20 years
□ >20 years

*Indicate how much the following parameters influence your determination of the transfusion strategy for a transfusion dependent patient.*

2.1 Dyspnoe d’effort

□ No influence □ Some influence □ Medium influence □ Strong influence

2.2 Chronic fatigue

□ No influence □ Some influence □ Medium influence □ Strong influence

2.3 Quality of life

□ No influence □ Some influence □ Medium influence □ Strong influence

2.4 Self-sustainability of the patient

□ No influence □ Some influence □ Medium influence □ Strong influence

2.5 Mobility of the patient

□ No influence □ Some influence □ Medium influence □ Strong influence

2.6 Emotional state of the patient (e.g. fear, depression)

□ No influence □ Some influence □ Medium influence □ Strong influence

2.7 Angina pectoris

□ No influence □ Some influence □ Medium influence □ Strong influence

2.8 Heart failure

□ No influence □ Some influence □ Medium influence □ Strong influence

2.9 Pulmonary diseases (e.g. COPD, Asthma)

□ No influence □ Some influence □ Medium influence □ Strong influence

2.10 Kidney failure

□ No influence □ Some influence □ Medium influence □ Strong influence

2.11 Other disease/organ failure(indicate what, and the influence) ___________

□ No influence □ Some influence □ Medium influence □ Strong influence

2.12 Periferal vascular problems

□ No influence □ Some influence □ Medium influence □ Strong influence

2.13 Iron parameters (Ferritin >1000)

□ No influence □ Some influence □ Medium influence □ Strong influence

2.14 Prior transfusion reactions

□ No influence □ Some influence □ Medium influence □ Strong influence

2.15 Tendency to bleed (possible better hemostasis after transfusion)

□ No influence □ Some influence □ Medium influence □ Strong influence

2.16 Adverse long term effect of RBC transfusions (e.g. iron overload, alloantibodies)

□ No influence □ Some influence □ Medium influence □ Strong influence

2.17 Vital parameters: Blood pressure, Heart rate, Saturation, Respiratory rate

□ No influence □ Some influence □ Medium influence □ Strong influence

2.18 Patient activity

□ No influence □ Some influence □ Medium influence □ Strong influence

2.19 Patient cognition: Effect of transfusion on a patients’ sustained attention

□ No influence □ Some influence □ Medium influence □ Strong influence

2.20 Patients’ sex

□ No influence □ Some influence □ Medium influence □ Strong influence

*Please fill out this part for a patient you are currently treating. If you have more transfusion dependent patient, you are encouraged to fill out more than one set of questions.*

3.1 Age of patient
□ 18-29
□ 30-39
□ 40-49
□ 50-59
□ 60-69
□ 70-79
□ 80-89
□ 90+

3.2 Sex ___________

3.3 Disease causing the chronic anaemia
□ Aplastic anaemia
□ MDS
□ MPN
□ Sickle cell disease (exchange transfusions excluded)
□ Thalassemia
□ Other: ___________

3.4 What is the transfusion trigger of this patient?

___________

3.5 How much RBC units do you transfuse to the patient when the Hb is <0.5 mmol/L below the trigger?

___________

3.6 How much RBC units do you transfuse to the patient when the Hb is 0.5-1.0 mmol/L below the trigger?

___________

3.7 How much RBC units do you transfuse to the patient when the Hb is >1.0 mmol/L below the trigger?

___________

3.8 What is the patients’ transfusion interval?
□ every week
□ every two weeks
□ every three weeks
□ every four weeks
□ every five weeks
□ every six weeks
□ every seven weeks
□ every eight weeks
□ other ___________

3.9 Does the patient receive erytropoietin stimulating agents?
□ Yes
□ No

3.10 Does the patient suffer from angina pectoris?
□ No
□ Very mild
□ Mild
□ Medium
□ Severe
□ Very severe

3.11 Does the patient suffer from cardiac failure?
□ No
□ Very mild
□ Mild
□ Medium
□ Severe
□ Very severe

3.12 Does the patient suffer from pulmonary problems?
□ No
□ Very mild
□ Mild
□ Medium
□ Severe
□ Very severe

3.13 Does the patient receive active treatment?
□ No
□ Epigenetic treatment
□ Immunomodulating agents
□ Chemotherapy

3.14 Does the patient suffer from another co-morbidity that influences the transfusion strategy?
□ No

□ Yes ___________
□ Very mild
□ Mild
□ Medium
□ Severe
□ Very severe

3.15 Does this patient get furosemide with a transfusion?
□ Yes, always
□ When >2 units are transfused
□ When >3 units are transfused
□ When >4 units are transfused
□ No, never

3.16 Does the patient receive chelation therapy
□ Yes, adequately
□ Yes, inadequately due to side effects
□ Yes, maximum dose, insufficient results
□ No, due to side effects
□ No, not required

3.17 What is the most recent ferritin of the patient? ___________

3.18 Does the patient have irregular blood group antibodies?
□ Yes
□ No

3.19 Does the patient have an increased bleeding tendency?
□ Yes
□ No

3.20 Do you have more tha one chronic transfusion dependent patient?
□ Yes 🡪 *Repeat questions 3.1 -3.20*
□ No
